# Draft genome sequence of multidrug-resistant *Raoultella ornithinolytica* isolated from a wastewater treatment plant in Laramie, Wyoming

**DOI:** 10.1128/mra.01051-25

**Published:** 2025-12-15

**Authors:** Puja Boidya, Anahita Ghorbani Tajani, Nicolas Blouin, Bledar Bisha

**Affiliations:** 1 Department of Animal Science, University of Wyoming 4416, Laramie, Wyoming, USA; 2 INBRE Data Science Core, University of Wyoming 4416, Laramie, Wyoming, USA; Fluxus Inc., Sunnyvale, California, USA

**Keywords:** antimicrobial resistance, *Raoultella ornithinolytica*, public health, wastewater

## Abstract

We report the whole-genome sequence of the multidrug-resistant *Raoultella ornithinolytica* strain w67, isolated from wastewater influent in Laramie, Wyoming, USA. The genome harbors *bla*_CTX-M-15_, *bla*_OXA-1_,*bla*_TEM-1_, along with additional antibiotic resistance genes and multiple virulence factors, offering genomic insights into the resistance mechanisms and pathogenic potential.

## ANNOUNCEMENT

*Raoultella ornithinolytica* (*R. ornithinolytica*) is a gram-negative, encapsulated member of the Enterobacteriaceae family, commonly found in soil, water, and plants (1, 2). Previously considered nonpathogenic, it is now recognized as an opportunistic pathogen associated with bacteremia as well as urinary tract infections and biliary tract infections (2–4). Certain strains harbor antimicrobial resistance (AMR) genes on mobile genetic elements, enabling horizontal gene transfer (5). Here, we report the first draft genome sequence of *R. ornithinolytica* strain w67 isolated from wastewater influent in Laramie, Wyoming, revealing multiple clinically relevant AMR genes, plasmids, and virulence factors underscoring its role in resistance dissemination.

*R. ornithinolytica* strain w67 was isolated from a 24-hour composite influent sample collected aseptically from the Laramie Wastewater Treatment Plant (WWTP) (41°20′09.2″ N, 105°36′04.5″ W). Samples were transported on ice and processed within 1 hour. Serial 10-fold dilutions were plated on MacConkey agar containing cefotaxime (1 µg/mL; MilliporeSigma, USA), yielding lactose-fermenting mucoid colonies after 24 h at 37 °C. Species-level identification for *R. ornithinolytica* was confirmed using matrix-assisted laser desorption/ionization time-of-flight mass spectrometry (Bruker Biotyper; score ≥2.2). Strain w67 was purified by three successive subcultures before antimicrobial susceptibility testing (AST). AST was performed by broth microdilution using 14-antibiotic pre-coated plates (CMV3AGNF, ThermoFisher Scientific, USA), and minimum inhibitory concentrations were interpreted after 18–24 hour with SWIN software v3.3 (ThermoFisher Scientific, USA) following CLSI guidelines (6).

Strain w67 was grown on MacConkey agar at 37°C for 24 h. A loopful (10 µL) of cells was removed from a single colony on agar, then genomic DNA was extracted using the DNeasy Blood & Tissue Kit (Qiagen, USA) and quantified with a Qubit 4.0 Fluorometer (Thermo Fisher Scientific, USA). DNA with a concentration ≥100 ng was processed using the Illumina DNA Prep kit (Illumina, USA). DNA was fragmented to ~350 bp with bead-linked transposomes, and adapters were added during tagmentation. Five PCR cycles incorporated dual i5/i7 indices, followed by bead-based purification. Library quality and fragment distribution were assessed using a QIAxcel system (Qiagen, USA). Equimolar libraries were pooled, diluted to 750 pM with RSB+Tween buffer, and sequenced on an Illumina NextSeq 2000 platform.

Sequencing quality was assessed with FastQC v0.12.1 (7) as previously described (8). Raw reads were trimmed using Trimmomatic v0.39 (9), assembled with SPAdes v4.0.0 (10), and annotated via PGAP v6.10 (11). Antibiotic resistance genes (ARGs) were detected with ABRicate v0.2.0 (12), virulence genes via VirulenceFinder v2.0 (13), and plasmids using PlasmidFinder v2.0.1 (14). Analyses were performed using default parameters unless specified otherwise.

We report the draft genome of strain w67, selected for its multidrug-resistant (MDR) profile and presence of diverse ARGs, plasmid replicons, and virulence factors. Sequencing identified 15 acquired ARGs, 8 of which corresponded to the observed resistance phenotypes (Table 1). These results emphasize the role of WWTPs as reservoirs of MDR *R. ornithinolytica*, highlighting their importance in AMR surveillance and public health risk assessment.

**TABLE 1:** Sequencing summary, phenotypic, and genomic characteristics features of *Raoultella ornithinolytica* strain w67 isolated from the Laramie WWTP influent

Characteristics	Genomic, Phenotypic, and Resistome Attributes
Draft Genome size (bp)	5,746,342
GC content (%)	55.52
Total reads (paired end)	3,551,348
Sequencing depth (×)	89.29×
No. of contigs	144
Largest contig (bp)	537,442
N50 / L50	326,504 bp / 7
Average contig length (bp)	39,905
Genes (total)	5,526
Coding sequences (CDS)	5,446 (including 5,367 protein-coding genes)
RNA genes	80
Phenotypic resistance	Resistant: AMP (ampicillin), XNL (ceftiofur), AXO (ceftriaxone), CIP (ciprofloxacin), GEN (gentamicin), TET (tetracycline), SXT (trimethoprim-sulfamethoxazole) Intermediate: AUG2 (amoxicillin-clavulanic) Susceptible: AZI (azithromycin), FOX (cefoxitin), CHL (chloramphenicol), NAL (nalidixic acid), FIS (sulfisoxazole)
Acquired ARGs	*oqxB9*, *oqxA10*, *qnrB1*, *tet(A*), *sul2*, *aph(3'')-Ib*, *aph(6)-Id*, *bla*_TEM-1_, *bla*_CTX-M-15_, *dfrA14*, *aac(3)-IIe*, *bla*_ORN-1_, *aac(6')-Ib-D181Y, bla*_OXA-1_, and *fosA*.
Plasmid replicons	IncFIB(K)_1_Kpn3, IncFII_1_pKP91, and IncFIA(HI1)_1_HI1.
Virulence genes	*ybtQ*, *ybtP*, *ybtA*, *irp2*, *irp1*, *ybtU*, *ybtT*, *ybtE*, *fyuA,* and *ompA*

## Data Availability

The draft genome assembly has been deposited in GenBank under the accession number JBPVPL000000000. The associated BioProject and BioSample identifiers are PRJNA1295580 and SAMN50178694, respectively. Raw sequencing reads are available in the Sequence Read Archive (SRA) under the accession number (SRR35248090).
